# Association between Thalamocortical Functional Connectivity Abnormalities and Cognitive Deficits in Schizophrenia

**DOI:** 10.1038/s41598-019-39367-z

**Published:** 2019-02-27

**Authors:** Pinhong Chen, Enmao Ye, Xiao Jin, Yuyang Zhu, Lubin Wang

**Affiliations:** Institute of Military Cognitive and Brain Sciences, Academy of Military Medical Sciences, Beijing, 100850 China

## Abstract

Cognitive deficits are considered a core component of schizophrenia and may predict functional outcome. However, the neural underpinnings of neuropsychological impairment remain to be fully elucidated. Data of 59 schizophrenia patients and 72 healthy controls from a public resting-state fMRI database was employed in our study. Measurement and Treatment Research to Improve Cognition in Schizophrenia (MATRICS) Battery was used to measure deficits of cognitive abilities in schizophrenia. Neural correlates of cognitive deficits in schizophrenia were examined by linear regression analysis of the thalamocortical network activity with scores of seven cognitive domains. We confirmed the combination of reduced prefrontal-thalamic connectivity and increased sensorimotor-thalamic connectivity in patients with schizophrenia. Correlation analysis with cognition revealed that in schizophrenia (1) the thalamic functional connectivity in the bilateral pre- and postcentral gyri was negatively correlated with attention/vigilance and speed of processing (Pearson’s *r* ≤ −0.443, *p* ≤ 0.042, FWE corrected), and positively correlated with patients’ negative symptoms (Pearson’s *r* ≥ 0.375, *p* ≤ 0.003, FWE corrected); (2) the thalamic functional connectivity in the right cerebellum was positively correlated with speed of processing (Pearson’s *r* = 0.388, *p* = 0.01, FWE corrected). Our study demonstrates that thalamic hyperconnectivity with sensorimotor areas is related to the severity of cognitive deficits and clinical symptoms, and extends our understanding of the neural underpinnings of “cognitive dysmetria” in schizophrenia.

## Introduction

Cognitive deficits among individuals with schizophrenia have been widely investigated, and are considered a core component of schizophrenia^1^. The cognitive deficits in patients with schizophrenia are robust and extensive, with a 1.5 to 2.5 standard deviation gap between patients and controls in all ability domains, as measured by standard neurocognitive tasks^2^. These impairments are present at the first-psychotic episode^3^ and clinically remitted states^4^, and schizophrenia patients who were never exposed to neuroleptics also show highly significant neuropsychological deficits^5^. The cognitive deficits are regarded as an important predictor of functional outcome for schizophrenia patients^6,7^, and are also found among other first-degree relatives of schizophrenia probands^8,9^. Although these studies provide important information regarding cognitive deficits as a potential precursor and vulnerability factor of schizophrenia, little is known about the neuropathological mechanisms underlying this core symptom across psychoses.

The thalamus, a subcortical structure that transmits information from the peripheral sensory nervous system to the cortex, has been recently targeted in schizophrenia research. By examining interregional correlations in spontaneous, low-frequency, blood-oxygen-level-dependent (BOLD) signal fluctuations, previous resting-state functional magnetic resonance imaging (fMRI) studies showed the importance of the abnormal thalamocortical functional interactions in the pathophysiology of schizophrenia^10^. In particular, these studies have consistently found the combination of decreased thalamic connectivity with the prefrontal cortex (PFC) and an increased connectivity with motor and somatosensory cortical areas in schizophrenia^2^. Recently, the reduced prefrontal-thalamic connectivity and sensorimotor-thalamic hyperconnectivity confirmed in a majority of chronic patients was also observed in the early stage of psychosis^11^ and clinical high-risk (CHR) individuals^12^, suggesting that thalamic dysconnectivity presents very early or even prior to the onset of illness. Congruous with these results, diffusion tensor imaging studies have proven that schizophrenia patients showed reduced structural connectivity of the thalamus to the lateral PFC, providing anatomical evidence for functional abnormalities of the thalamus in schizophrenia^13^.

Recently, there is increasing evidence to suggest that abnormalities of the thalamocortical network may contribute to cognitive deficits in individuals with schizophrenia. Indeed, imaging studies have shown that decreased activation of the thalamus in schizophrenia patients during working memory tasks^14^, and that the reduced prefrontal-thalamic connectivity in patients correlated with impaired working memory^15^. On the contrary, Seidman *et al*. found that relatives of schizophrenia patients showed significantly greater task-elicited activation in the right dorsomedial thalamus, when carrying out an auditory continuous performance test^16^. Another study also proved significant hyperactivation in the thalamus/basal ganglia of patients for both verbal and spatial working memory tasks^17^. Animal models have also proven that a subtle decrease in mediodorsal (MD) thalamus activity is enough to cause impairment, particularly in cognitive tasks such as reversal learning and working memory^18^. Additionally, Schmitt *et al*. found that MD thalamus stimulation amplifies prefrontal functional connectivity and promotes performance in forced-choice tests of attentional control^19^. However, the specific effect of different thalamocortical circuits on cognitive deficits in schizophrenia remains largely unknown.

To search for neural mechanisms underlying clinical and cognitive symptoms in schizophrenia, we investigated the change of functional interactions between different cortical areas and the thalamus, and its association with cognitive deficits in schizophrenia. Parceling the cortex into six regions of interest^20^, functional connectivity between cortical ROIs and thalamic ROIs were compared between control and patient groups. Then, we employed the MATRICS Battery to examine the relationship between different thalamocortical circuits and a range of key cognitive constructs in schizophrenia patients. The MATRICS evaluates seven dimensions of cognitive performance, including attention/vigilance, problem solving, speed of processing, social cognition, verbal learning, visual learning and working memory, and is a standard tool for assessing cognition in cognitive remediation strategies in clinical trials^21^. Based on previous studies, we hypothesized that impaired cognitive function in schizophrenia might be related to the reduced prefrontal-thalamic connectivity as well as increased sensorimotor-thalamic connectivity.

## Results

### Demographic, clinical and cognitive results

Sample demographics are shown in Table 1. There was no significant difference in age (healthy subjects: 35.86 ± 11.67 years; psychosis: 37.69 ± 13.77; *t* = 0.81, *p* = 0.419) and smoking status (healthy subjects: 21.1% smokers; psychosis: 33.9% smokers; *χ*^2^_1_ = 2.67, *p* = 0.102) between schizophrenia patients and healthy subjects. The two groups showed marginal significance in sex ratio (healthy subjects: 68.1% male subjects; psychosis: 83.1% male subjects; *χ*^2^_1_ = 3.87, *p* = 0.049), and the WASI FSIQ (four subtests) in the schizophrenia group was significantly lower than that in the healthy group (healthy subjects: 111.79 ± 11.92; psychosis: 100.89 ± 16.87; *t*_119_ = −4.10, *p* < 0.001). The age at onset of schizophrenia was 22.10 ± 8.74 years, and the average duration of illness was 15.59 ± 12.09 years. PANSS positive and negative and general psychopathology scores in schizophrenic patients were 14.66 ± 4.59, 14.81 ± 5.03, and 29.12 ± 8.02 respectively. The average daily dose of antipsychotics, factoring in chlorpromazine equivalents established in Gardner *et al*.^22^, was 369.25 ± 318.69 mg/d in schizophrenia patients.

**Table 1:** Sample Demographics.

Characteristic	Healthy Subjects	Schizophrenia Patients	Statistics	
	*n* = 72	*n* = 59	*χ* ^2^ */t*	*p*
Gender (Male: female)	49:23	49:10	3.87	**0.049**
Smoking Status (Smoker: Non-smoker)	15:56	20:39	2.67	0.102
Age (years)	35.86 ± 11.67	37.69 ± 13.77	0.81	0.419
WTAR T-score	109.02 ± 13.02	102.71 ± 13.50	−2.59	**0.011**
WASI VIQ	106.75 ± 11.22	99.29 ± 16.67	−2.93	**0.004**
WASI PIQ	114.23 ± 12.43	103.89 ± 16.23	−3.88	** < 0.001**
WASI FSIQ (four subtest)	111.79 ± 11.92	100.89 ± 16.87	−4.10	** < 0.001**
Age at First Illness		22.1 ± 8.74		
Duration of Illness (years)		15.59 ± 12.09		
PANSS Positive		14.66 ± 4.59		
PANSS Negative		14.81 ± 5.03		
PANSS General		29.12 ± 8.02		
CPZ Equivalents		369.25 ± 318.69		

WTAR, Wechsler Test of Adult Reading; Wechsler Abbreviated Scale of Intelligence (WASI); VIQ, verbal intelligence; PIQ, performance intelligence; FSIQ, full scale intelligence; PANSS, Positive and Negative Syndrome Scale; CPZ, chlorpromazine; *p* values < 0.05 are labelled in bold.

**Figure 1:**
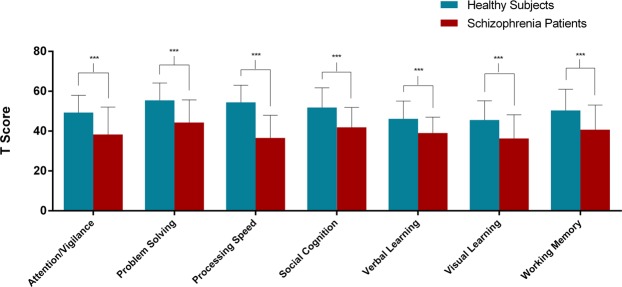
Significantly decreased MATRICS performance in schizophrenia group compared to control group. The results suggested that patients exhibited significantly lower scores on all the cognitive domains of MATRICS. ***Indicates *p* < 0.001. Error bars represent standard deviation.

As presented in Fig. 1, patients exhibited significantly lower scores on all the cognitive domains of MATRICS compared to control subjects (*t*-tests, the range of all *t*-values −4.58~−9.56, all *p* values were < 0.001). Group means and standard deviations for performance scores of individual tests of MATRICS are shown in Supplementary Table S1. To confirm that the results were not affected by patients’ lower premorbid and current intellect, we performed covariance analysis controlling for these effects on cognition. Results indicated that, compared with healthy subjects, patients still showed significantly decreased cognition on all the cognitive domains of MATRICS.

### Altered thalamocortical functional connectivity in patients

**Figure 2:**
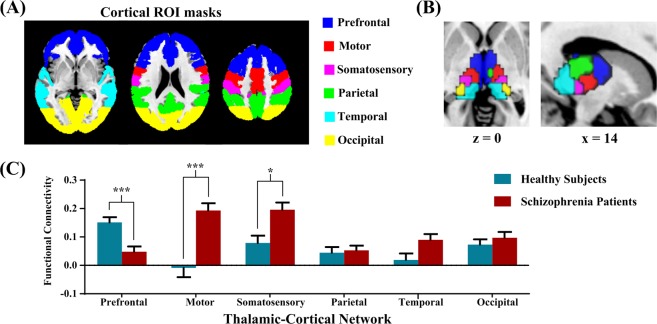
Thalamo-cortical functional connectivity in schizophrenia and healthy subjects. (**A**) Six cortical ROI masks were overlaid onto the standard MNI brain. (**B**) Functional thalamic subdivision was created using the winner-takes-all strategy, in which each thalamic voxel was labelled according to the cortical ROI with the highest connectivity strength. (**C**) Significantly altered thalamocortical connectivity in schizophrenia patients compared to healthy subjects. The patient group showed decreased PFC-thalamic functional connectivity and increased motor-thalamic and somatosensory-thalamic connectivity. *Indicates *p* < 0.05 and ***Indicates *p* < 0.001. Error bars represent standard error.

According to prior studies^2^, we parceled the thalamus into six functional subregions based on their functional connectivity with the cortical ROIs (Fig. 2A). As shown in Fig. 2B, the cortical ROI-to-thalamic ROI analysis demonstrated that each cortical region of interest was connected to distinct, largely non-overlapping divisions of the thalamus. Group differences in thalamocortical connectivity, correcting by Bonferroni adjustment, are depicted graphically in Fig. 2C. Compared with healthy subjects, the patient group exhibited weaker PFC-thalamic network connectivity (*p* < 0.001), and stronger motor-thalamic and somatosensory-thalamic network connectivity (*p* < 0.001 and *p* = 0.002, respectively). No significant differences were observed between groups in parietal, temporal and occipital cortex connectivity with the thalamus.

**Figure 3:**
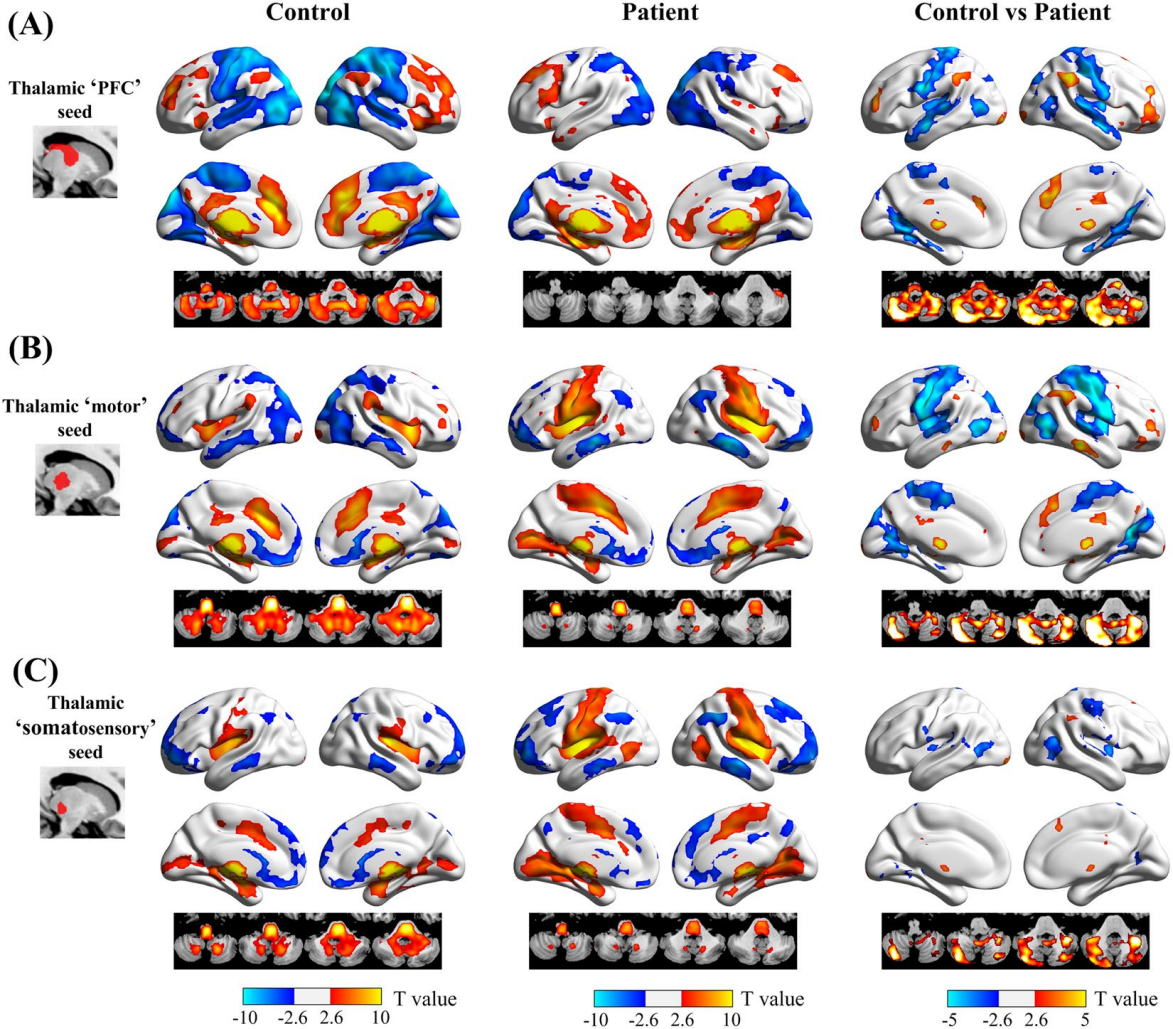
Functional dysconnectivity of the PFC, motor, and somatosensory thalamus seeds in schizophrenia. For the thalamic PFC (**A**) and motor (**B**) seeds, patients with schizophrenia showed reduced functional connectivity with the dorsolateral PFC, dorsal anterior cingulate cortex, inferior parietal lobule, and cerebellum (warm colors), and increased functional connectivity with the pre- and postcentral gyri, superior and middle temporal gyri, lateral and medial occipital regions (cold colors), relative to healthy subjects. In contrast, functional connectivity of the thalamus somatosensory seed (**C**) was mainly restricted to the postcentral gyrus and lateral occipital region.

We further conducted seed-based analyses using the thalamic PFC, motor, and somatosensory subregions as seeds to better localize thalamocortical network abnormalities in patients with schizophrenia. The resting-state functional connectivity patterns of different thalamic subregions are shown in Fig. 3A–C, respectively. We can see that the thalamic PFC seed was positively correlated with brain regions in the executive control network, including the dorsolateral PFC, inferior frontal gyrus/anterior insula, anterior and middle cingulate cortices, and inferior parietal lobule, and was negatively correlated with the occipital and sensorimotor cortices. In contrast, the thalamic motor and somatosensory seeds were positively correlated with the primary and secondary motor and somatosensory cortices, and negatively correlated with the medial PFC, middle temporal gyrus, and lateral occipital lobe.

Direct comparison between healthy subjects and schizophrenia patients showed differences in thalamocortical functional connectivity between groups in specific brain regions. For the thalamic ‘PFC’ seed, schizophrenia patients exhibited significantly decreased positive functional connectivity in the dorsolateral PFC, dorsal anterior cingulate cortex, inferior parietal lobule, and cerebellum. Patients also showed decreased negative functional connectivity in the pre- and postcentral gyri, superior and middle temporal gyri, and the lateral and medial occipital regions upon evaluation of the thalamic PFC seed. For the thalamic ‘motor’ seed, schizophrenia patients exhibited significantly increased positive functional connectivity in the pre- and postcentral gyri and lateral and medial occipital regions, decreased positive functional connectivity in the dorsal anterior cingulate cortex and cerebellum, and increased negative functional connectivity in the dorsolateral PFC and inferior parietal lobule.

### Cognitive correlates of thalamocortical dysconnectivity

Linear regression analyses were performed to examine the relationships between thalamocortical functional connectivity and specific cognitive functions in patients.

**Figure 4:**
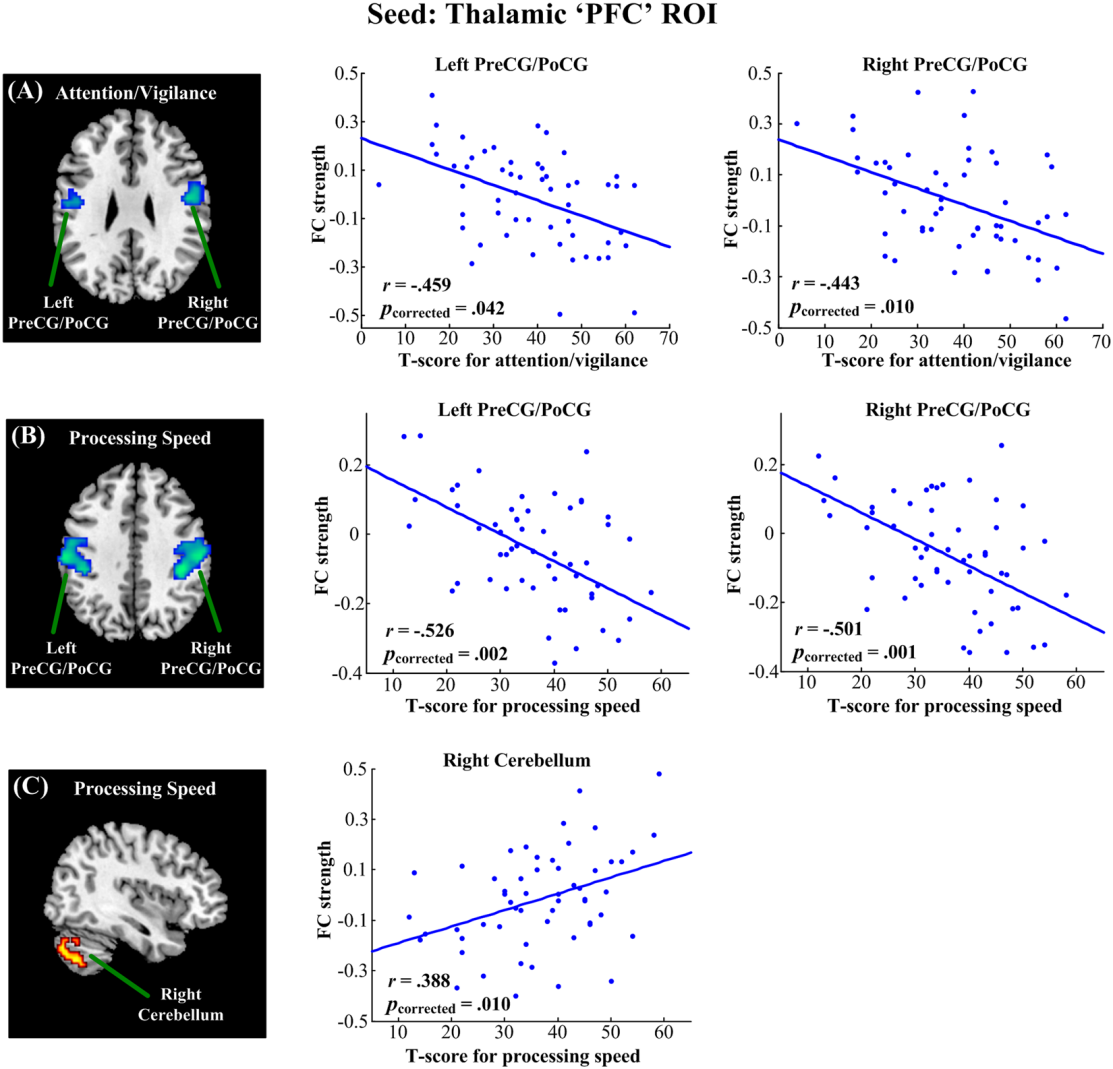
Linear regression analysis between the functional connectivity of the thalamic ‘PFC’ ROI and MATRICS domain scores in schizophrenia. Attention/vigilance was negatively correlated with functional connectivity in the bilateral PreCG/PoCG (**A**); speed of processing was positively correlated with functional connectivity in the right cerebellum (**B**) and negatively correlated with functional connectivity in the bilateral PreCG/PoCG (**C**). All results were thresholded at *p* < 0.05, corrected for multiple comparisons with family wise error (FWE). PreCG, precentral gyrus; PoCG, postcentral gyrus.

As shown in Fig. 4, using thalamic PFC subregion as the seed, we found that attention/vigilance was negatively correlated with functional connectivity in the bilateral pre- and postcentral gyri (left side: Pearson’s *r* = −0.459, *p* = 0.042; right side: Pearson’s *r* = −0.443, *p* = 0.010; FWE corrected). Moreover, the speed of processing was positively correlated with functional connectivity in the right cerebellum (Pearson’s *r* = 0.388, *p* = 0.010, FWE corrected), and negatively correlated with functional connectivity in the bilateral pre- and postcentral gyri (left side: Pearson’s *r* = −0.526, *p* = 0.002; right side: Pearson’s *r* = −0.501, *p* = 0.001; FWE corrected).

**Figure 5:**
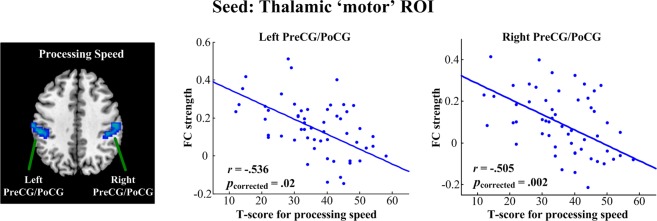
Linear regression analysis between the functional connectivity of thalamic ‘motor’ ROI and MATRICS domain scores in schizophrenia. Speed of processing was negatively correlated with functional connectivity in the PreCG/PoCG. All results were thresholded at *p* < 0.05, corrected for multiple comparisons with family wise error (FWE). PreCG, precentral gyrus; PoCG, postcentral gyrus.

When using the thalamic motor subregion as a seed (Fig. 5), we found that the speed of processing was negatively correlated with functional connectivity in the bilateral pre- and postcentral gyri (left side: Pearson’s *r* = −0.536, *p* = 0.02; right side: Pearson’s *r* = −0.505, *p* = 0.002; FWE corrected).

### Symptom correlates of thalamocortical dysconnectivity

**Figure 6:**
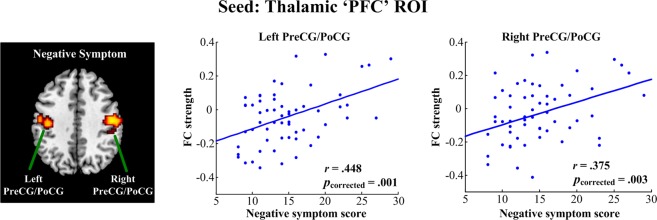
Linear regression analysis between the functional connectivity of the thalamic ‘PFC’ ROI and symptoms in schizophrenia. Patients’ negative symptoms were positively correlated with functional connectivity in the PreCG/PoCG. All results were thresholded at *p* < 0.05, corrected for multiple comparisons with family wise error (FWE). PreCG, precentral gyrus; PoCG, postcentral gyrus.

When using thalamic PFC subregion as seed (Fig. 6), we found that patients’ negative symptoms were positively correlated with functional connectivity in the bilateral pre- and postcentral gyri (left side: Pearson’s *r* = 0.448, *p* = 0.001; right side: Pearson’s *r* = 0.375, *p* = 0.003; FWE corrected). However, the remaining thalamocortical functional connectivity did not show any significant correlation to the severity of clinical symptoms.

### The effect of nuisance variables

We performed additional analyses to examine the relationship between thalamocortical functional connectivity and age, sex, duration of illness and medication. Among patients, we found that age was negatively correlated with functional connectivity between the thalamic ‘PFC’ subregion and the cerebellum (p < 0.001; FWE corrected); sex, duration of illness and medication did not correlate with functional connectivity of any thalamocortical networks (p > 0.05; FWE corrected).

## Discussion

Consistent with previous studies^2^, we demonstrated the combination of reduced prefrontal-thalamic and increased sensorimotor-thalamic functional connectivity in schizophrenia patients. Accordingly, our results revealed that lower cognition scores in schizophrenia correlated with both enhanced connectivity of the thalamus with sensorimotor network and a weakened connectivity of the thalamus with cerebellar regions. These findings may extend our understanding of the neural underpinnings of “cognitive dysmetria” of schizophrenia.

In this study, we found that cognitive deficits were mainly related to the functional connectivity of the thalamic ‘PFC’ subregion, which corresponded closely to the thalamic mediodorsal nucleus (MD). Consistent with our findings, Giraldo-Chica *et al*. demonstrated reduced structural connectivity between the lateral PFC and the MD thalamus, which positively correlated with scores of the Wechsler Memory Scale-III (WMS-III) Working Memory Index^15^. Previous animal studies have also proven that impairments in cognitive tasks, such as reversal learning and recognition memory, could be resulted from aberrant structural^23^ and functional^18^ changes of mediodorsal (MD) thalamus. In addition, some studies have implicated the role of MD thalamus in working memory and attentional control^19,24^. The MD thalamus shares reciprocal excitatory projections with the PFC^25,26^, making it a hub for causing exact impairments to cognitive functions^18^. Collectively, these studies consistently support that the thalamic ‘PFC’ subregion plays a key role in the cognitive symptoms of schizophrenia.

Our study showed that the thalamic functional connectivity with sensorimotor regions was negatively correlated with higher cognitive functions such as attention/vigilance and speed of processing. Imaging studies have demonstrated reduced activation of the sensorimotor cortices during a sequential finger tapping task^27^, which may contribute to the pathogenesis of soft neurological signs, a particular clinical sign for schizophrenia diagnosis^28,29^. Using a data-driven analysis of resting-state fMRI fluctuations, the connectivity between the visual and sensorimotor regions was found to be significantly decreased in the schizophrenia patients^30^. Berman *et al*. illustrated the negative correlation between sensorimotor and higher order regions and its link to positive symptoms in childhood-onset schizophrenia^31^. In addition, prefrontal-thalamic connectivity is developed and sensorimotor-thalamic connectivity is refined in the normal brain during the transition from adolescence to adulthood^32^. Therefore, irregular brain maturation might underline the neuropathological mechanisms of schizophrenia^20^. In short, the findings of our study may facilitate understanding of the cognitive deficits in patients with schizophrenia as a result of reduced prefrontal-thalamic connectivity and increased sensorimotor-thalamic connectivity.

In addition, we have found decreased connectivity of the cerebellum with the thalamic ‘PFC’ subregion, and its positive correlation with cognitive function in schizophrenia. The role of the cerebellum in schizophrenia has been highlighted by Andreasen’s hypothesis of ‘cognitive dysmetria’, which suggests that dysfunction in the cortico-cerebellar-thalamo-cortical circuit may underlie the widespread disturbances in information transmission and processing in schizophrenia^33,34^. Notably, some studies have reported abnormal functional connectivity associated with the cerebellum in schizophrenia^35,36^. It is also suggested that the dysfunction of the thalamus might act as a mediator in the synchronized activity between the cerebellum and prefrontal cortex in schizophrenia^37^. Based on previous findings, our study further indicates that cerebellar dysfunction could account for some of the psychiatric or cognitive symptoms present in this disease^38^.

We also found that patients’ negative symptoms were positively related to hyperconnectivity between the thalamus and sensorimotor cortex. A functional connectivity study with autistic patients have found that the dysfunction of sensorimotor cortex might be associated with impairments of social ability and communication, including motivation for social engagement and emotion recognition^39^. It has also been proposed that basic perceptivo-motor deficits may be responsible for impaired facial and vocal emotion decoding in schizophrenia^40^. Additionally, several studies have reported similar correlations between sensorimotor-thalamic hyperconnectivity and clinical symptoms in patients with schizophrenia^41,42^. However, other studies did not find any significant relationship^11,20^. These studies suggest that the clinical correlates of thalamocortical dysconnectivity in schizophrenia need to be further explored and better understood.

Our study has several limitations. First, the target nuclei of the thalamus are small compared to the cortical regions that are typically highlighted in resting-state functional connectivity analysis. The definition of thalamic subregions and their boundaries using conventional fMRI is generally coarse and tends to combine signals from adjacent structures. Recent advances in data acquisition methods, such as ultrahigh speed and ultrahigh field fMRI^43^, may provide more accurate estimations of thalamocortical networks. Second, although we did not find any evidence that antipsychotic treatment was associated with functional dysconnectivity in schizophrenia, it is possible that medication effects on connectivity may not be dose-dependent. In addition, functional dysconnectivity may be associated with long term hospitalization and social isolation due to disease chronicity. Studies exploring thalamic dysconnectivity in nonmedicated first-episode psychosis are required to exclude the effect of illness stages. Finally, in future studies, we intend to combine fMRI with DTI to illustrate how functional dysconnectivity of the thalamus is linked with abnormalities in anatomical connectivity in schizophrenia.

In summary, the findings of our study suggest that the unbalanced thalamic networks make a critical contribution to damaged cognition that may underlie the neuropathological mechanisms in schizophrenia. This further facilitates our understanding of thalamocortical dysconnectivity as a biomarker of cognitive deficits and as a neural intervention target.

## Methods

### Participants

An open access resting-state fMRI dataset of schizophrenia patients and healthy subjects, formerly studied by different authors^2^, was employed in our study. This publicly available dataset is maintained by the Center for Biomedical Research Excellence (COBRE, http://fcon_1000.projects.nitrc.org/indi/retro/cobre.html). Patients were recruited from the outpatient clinic at the University of New Mexico Health Sciences Center. A psychiatrist made judgments about whether patients were clinically stable and appropriate for the study. Participants were administered the Structured Clinical Interview for the Diagnostic and Statistical Manual of Mental Disorders, 4th edition (DSM-IV SCID–Clinician Version)^44^ by a trained research assistant, and the interview process was supervised by either F. M. Hanlon or J. R. Bustillo^45^. The patients’ exclusion criteria were as follows: history of neurological disorder, mental retardation, severe head trauma with more than 5-minute loss of consciousness, and substance abuse or dependence within the last 12 months. The controls’ exclusion criterion was having any psychiatric dysfunction. Informed consent was obtained from all subjects, according to institutional guidelines required by the Institutional Review Board at the University of New Mexico. All experiments were performed in accordance with relevant guidelines and regulations.

### Neuropsychological Measures

Premorbid intelligence was assessed by the Wechsler Test of Adult Reading (WTAR)^46^ and intellectual functioning was assessed by the Wechsler Abbreviated Scale of Intelligence (WASI)^47^, a short and reliable measure of intelligence. Of the WASI subtests, the Vocabulary and Similarities tests were used to estimate verbal IQ (VIQ), Block Design and Matrix Reasoning were used to estimate performance IQ (PIQ), and full-scale IQ (FSIQ) was computed by averaging the PIQ and VIQ scores. The MATRICS consensus cognitive battery^48^ consists of seven separable factors, including attention/vigilance, problem solving, speed of processing, social cognition, verbal learning, visual learning, and working memory. Here, t-scores were used for MATRICS, and lower scores indicated greater impairments in cognitive functions. Additionally, patients were also assessed with the Positive and Negative Syndrome Scale (PANSS) to quantify the severity of psychosis symptoms^49^.

### fMRI Data Acquisition and Preprocessing

The subjects underwent resting-state scans in a 3-T SIEMENS MRI scanner with the following parameters: 33 axial slices, repetition time = 2000 ms, echo time = 29 ms, flip angle = 75°, slice thickness = 3.5 mm, slice gap = 1.05 mm, acquisition matrix = 64 × 64, field of view = 240 mm. The total scan lasted 5 min, and 150 volumes of functional images were obtained. More information about the data acquisition can be found at http://coins.mrn.org/dx.

Data preprocessing was performed using SPM8 software (Wellcome Department of Imaging Neuroscience, University College London, UK; http://www.fil.ion.ucl.ac.uk/spm). Prior to preprocessing, the first 5 volumes of each scan were discarded due to magnetic saturation. Images were normalized to the standard EPI template in the MNI space and were resliced to 3 × 3 × 3 mm^3^. The resulting images were spatially smoothed with a Gaussian filter of 6 mm full-width half-maximum kernel. For functional connectivity analyses, several additional de-noising steps were conducted to reduce spurious variance that was unlikely to reflect neuronal activity. These steps included: (1) removal of nuisance signals from the data via multiple regression, including signals averaged from white matter, cerebrospinal fluid, and the whole brain, and the six parameters obtained by head motion correction, (2) temporal filtering with a band-pass filter (0.01–0.1 Hz), and (3) linear detrending to remove any residual drift.

Head motion has been increasingly found to be a serious factor that confounds estimation of functional connectivity^50,51^. Here, we used the artifact detection tool (ART; http://www.nitrc.org/projects/artifact_detect) to identify motion outliers. An image was defined as an outlier if the absolute head motion was >1 mm from previous scan, or if the scan-to-scan global signal change was >3 standard deviations of the global brain signal for the entire resting scan. Subjects with serious head motion (>20% outlier volumes) were excluded from this study, leaving a final sample of 72 healthy subjects and 59 schizophrenia patients. Outliers in the global mean signal intensity and motion were scrubbed by including them as nuisance regressors (i.e. one regressor per outlier) during the de-noising procedure. After scrubbing, residual head motion was still significantly different between groups (control: 0.25 ± 0.11 mm; patient: 0.30 ± 0.13 mm; two-sample t-test, *p* = 0.024). To minimize potential bias caused by subtle motion, residual head motion was included as a nuisance covariate in the statistical comparisons.

### Functional connectivity analysis

First, we compared functional connectivity between anatomically defined cortical ROIs and functionally defined thalamic ROIs across groups. As described previously^20,52^, the cortex was divided into six non-overlapping ROIs corresponding to the main targets of specific thalamic nuclei (the PFC, motor cortex, somatosensory cortex, posterior parietal cortex, temporal lobe and occipital lobe). The cortical ROI and thalamus masks were separately defined in standard MNI space by using the Harvard-Oxford cortical and subcortical probabilistic atlases in FSL (http://fsl.fmrib.ox.ac.uk/fsl/fslwiki/Atlases). A 25% threshold was applied to these atlases to avoid partial inclusion of neighboring ventricles. Figure 2A shows the six cortical ROI masks, which are equivalent to those used in Woodward *et al*.^20^. The Pearson’s correlation coefficient was then computed between the average BOLD signals from each of the cortical ROIs and BOLD signals from every voxel in the thalamus. Fisher’s z-transformation was applied to the correlation values to ensure normality. Based on the entire dataset of 131 subjects, functional thalamic subdivision was created using the winner-takes-all strategy, in which each thalamic voxel was labeled according to the cortical ROI with the highest connectivity strength^11,52^. For each participant, six thalamocortical network values were calculated by averaging connectivity strength of the voxels within each thalamic functional ROI with its respective cortical ROI, which served as the dependent variable in the cortical ROI-to-thalamic ROI analysis.

Second, seed-based analyses examining connectivity of functionally defined thalamic subregions with the rest of the brain were performed to better localize thalamocortical network abnormalities in patients with schizophrenia. Briefly, for each subject and for each thalamic ROI, the correlation map was created by calculating the Pearson’s correlation coefficients between the average BOLD signal of the thalamus ROI and BOLD signals of all voxels in the brain. These correlation maps were then converted to Z-value maps using Fisher’s r-to-z transformation.

### Statistical analysis

Behavioral data and cortical ROI-to-thalamic ROI functional connectivity data were compared between the control and schizophrenia groups using SPSS (version 20, IBM Inc, USA). To reduce the number of false positive or Type I error, the results were considered significant at *p* < 0.05, Bonferroni corrected for multiple comparisons replaced the nominal significance level *α* with the level *α/k* for each test (*k* is the number of tests being performed).

Voxel-wise statistical analyses were carried out using the general linear model in SPM8. First, correlation maps of control and schizophrenia subjects separately underwent a two-tailed, one-sample t-test, to determine brain regions with significant positive or negative correlations with each thalamic ROI. Then, a two-tailed, two-sample t-test was conducted to identify significant functional connectivity differences in the correlation maps of each thalamic ROI between the schizophrenia and control groups. The correlation analysis with the MATRICS domain scores across schizophrenia subjects was restricted to voxels within the masks, which were identified in the analysis between groups, to determine the association between thalamocortical functional connectivity abnormalities and cognitive deficits in schizophrenia. We also examined the correlations between thalamocortical functional connectivity and patients’ symptoms, including PANSS positive, negative and general. Each analysis was adjusted using nuisance covariates for age, sex and residual head motion. Duration of illness and CPZ equivalents were also used as nuisance covariates for the correlation analysis with cognition. The significance level was set at a cluster-level of *p* < 0.05, and data were corrected for multiple comparisons with the family wise error (FWE) and a voxel-level threshold of *p* < 0.005.

## Supplementary information

Supplementary Table S1. Group Means and Standard Deviations for Performance Scores of Individual Tests of MATRICS

## Data Availability

The datasets analyzed during the current study are available from the corresponding author upon reasonable request.
