# Supplementary material for: Association between Thalamocortical Functional Connectivity Abnormalities and Cognitive Deficits in Schizophrenia
Source: Sci Rep. 2019 Feb 27;9:2952. doi: 10.1038/s41598-019-39367-z (PMC6393449; doi:10.1038/s41598-019-39367-z)
Supplement: Supplementary file 1 — Supplementary Table S1. Group Means and Standard Deviations for Performance Scores of Individual Tests of MATRICS [file 41598_2019_39367_MOESM1_ESM.docx]

**Supplementary Information**

Association between Thalamocortical Functional Connectivity Abnormalities and Cognitive Deficits in Schizophrenia

Pinhong Chen^1^, Enmao Ye^1^, Xiao Jin^1^, Yuyang Zhu^1^, Lubin Wang^1,*^

^1^Institute of Military Cognitive and Brain Sciences, Academy of Military Medical Sciences, Beijing 100850, China

*****Corresponding Author**:** 27 Taiping Road, Beijing 100850; E-mail: wlbcc@126.com

**Supplementary Table S1.** Group Means and Standard Deviations for Performance Scores of Individual Tests of MATRICS

|  | Tests | Performance Scores | | | | Statistics | |
| --- | --- | --- | --- | --- | --- | --- | --- |
| Domain |  | Healthy Subjects(*n*=72) | | Schizophrenia Patients(*n*=59) | | *t* | *p* |
|  |  | Mean | SD | Mean | SD |  |  |
| Speed of Processing | TMT-A | 54.78 | 9.82 | 38.67 | 13.31 | -7.43 | **<.001** |
|  | BACS | 54.05 | 8.97 | 37.21 | 8.97 | -10.29 | **<.001** |
|  | Ani Flu | 51.42 | 8.22 | 42.38 | 12.93 | -4.65 | **<.001** |
| Attention/Vigilance | CPTIP | 49.47 | 8.93 | 38.18 | 13.95 | -5.15 | **<.001** |
| Working Memory | WMS-Ⅲ | 51.48 | 10.23 | 43.13 | 11.27 | -4.24 | **<.001** |
|  | LNS | 49.15 | 10.14 | 41.55 | 12.25 | -3.68 | **<.001** |
| Verbal Learning | HVLT | 46.06 | 8.93 | 39.02 | 8.03 | -4.57 | **<.001** |
| Visual Learning | BVMT | 45.60 | 9.62 | 36.21 | 11.97 | -4.78 | **<.001** |
| Reasoning and Problem Solving | NAB | 55.44 | 8.68 | 44.43 | 11.49 | -5.87 | **<.001** |
| Social Cognition | MSCEIT | 51.82 | 9.97 | 42.02 | 10.04 | -5.34 | **<.001** |

TMT-A, Trail Making Test, Part A; BACS, Brief Assessment of Cognition in Schizophrenia; Ani Flu, Animal Fluency; CPTIP, Continuous Performance Test-Identical Pairs; WMS-Ⅲ, Wechsler Memory Scale-Ⅲ; LNS, Letter-Number Span; HVLT, Hopkins Verbal Learning Test; BVMT, Brief Visuospatial Memory Test; NAB, Neuropsychological Assessment Battery; MSCEIT, Mayer-Salovey-Caruso Emotional Intelligence Test; *p* values <.05 are labeld in bold.
